# Correction to: Evaluating the efficacy and safety of human anti-SARS-CoV-2 convalescent plasma in severely ill adults with COVID-19: A structured summary of a study protocol for a randomized controlled trial

**DOI:** 10.1186/s13063-020-04504-x

**Published:** 2020-06-16

**Authors:** Christina M. Eckhardt, Matthew J. Cummings, Kartik N. Rajagopalan, Sarah Borden, Zachary C. Bitan, Allison Wolf, Alex Kantor, Thomas Briese, Benjamin J. Meyer, Samuel D. Jacobson, Dawn Scotto, Nischay Mishra, Neena M. Philip, Brie A. Stotler, Joseph Schwartz, Beth Shaz, Steven L. Spitalnik, Andrew Eisenberger, Eldad A. Hod, Jessica Justman, Ken Cheung, W. Ian Lipkin, Max R. O’Donnell

**Affiliations:** Columbia University Medical Center, New York, USA

**Correction to: Trials 21, 499 (2020)**

**https://doi.org/10.1186/s13063-020-04422-y**

Following publication of the original article [1], the authors identified an error in the author name of Andrew Eisenberger.

The incorrect author name is: Andrew Eisenberg

The correct author name is: Andrew Eisenberger

The author group has been updated above and the original article [1] has been corrected.
